# Visuo-spatial attention and semantic memory competition in the parietal cortex

**DOI:** 10.1038/s41598-023-33533-0

**Published:** 2023-04-17

**Authors:** Paolo Capotosto, Valentina Sulpizio, Gaspare Galati, Antonello Baldassarre

**Affiliations:** 1 Department of Neuroscience Imaging and Clinical Science, University “G. d’Annunzio”, Via dei Vestini 33, 66100 Chieti, Italy; 2 Brain Imaging Laboratory, Department of Psychology, Sapienza University, 00185 Rome, Italy; 3 Department of Cognitive and Motor Rehabilitation and Neuroimaging, Santa Lucia Foundation (IRCCS Fondazione Santa Lucia), Rome, Italy

**Keywords:** Neuroscience, Cognitive neuroscience, Learning and memory, Human behaviour

## Abstract

Neuroimaging studies associate specific functional roles to distinct brain regions investigating separate cognitive processes using dedicated tasks. For example, using both correlative (i.e., fMRI) and causal (i.e., TMS) approaches it has been shown the involvement of intra-parietal sulcus (IPS), as part of the dorsal attention network, in spatial attentional tasks as well as the importance of the angular gyrus (AG), as part of the default mode network, during the selection of relevant information in semantic memory. Nonetheless, in our daily life attention and semantic memory are rarely needed in isolation. In the present TMS study we investigate how the brain combines attentional and semantic memory demands in a single task. Results showed that, compared to a pseudo-TMS, stimulation of IPS, but not AG, affects behavioral performance, thus suggesting its preponderant role in such a combined task. Moreover, the lack of difference between the effect of IPS and AG stimulations seems to suggest that the two regions may be coactivated or that a third-party source might indirectly mediate the interaction between the two networks.

## Introduction

In the latest 15 years, a growing body of evidence has shown that the human cerebral cortex can be functionally segregated into a limited set of resting-state networks (RSNs), each composed of several nodes (regions) located in different lobes, which are characterized by coherent spatio-temporal patterns of spontaneous activity. Since these networks are usually studied in resting-state situations, their individual contribution to psychological states and mental processes has proven difficult to understand, and this network perspective has proven difficult to reconcile with the classical locationist view of the cerebral cortex which has dominated cognitive psychology.

In particular, the dichotomy between the so called “dorsal attention” (DAN) and “default mode” (DMN) networks, characterized by a competitive relationship with each other both at rest and during task performance^1^, has been often described in psychological terms as controlling external/environmental vs. internal/self-referential processes. For example, neuroimaging studies showed that, within the parietal cortex, the bilateral intraparietal sulcus (IPS), part of the DAN, is involved in visuo-spatial attention tasks^2^ Neuron)^3,4^. On the contrary, the left angular gyrus (AG, one of the main nodes of the DMN^5,6^, as well as of the language network^7^, is one of the cortical regions mostly engaged in internally oriented tasks^8^ including semantic^9,10^ and episodic^11^ memory.

Such correlative results have been causally confirmed by our group using a causal approach by combining transcranial magnetic stimulation (TMS) and electro-encephalography (EEG), showing that both behavioral performance and several local and global EEG markers (e.g., the parietal event related de-synchronization of the alpha rhythms and the topography of the EEG microstates) were selectively affected when TMS was delivered over the two parietal regions^12,13^. Specifically, stimulation of IPS, but not AG, interferes during the execution of a visuo-spatial attention task, whereas stimulation of AG, but not IPS, interferes during a semantic decision task.

**Figure 1:**
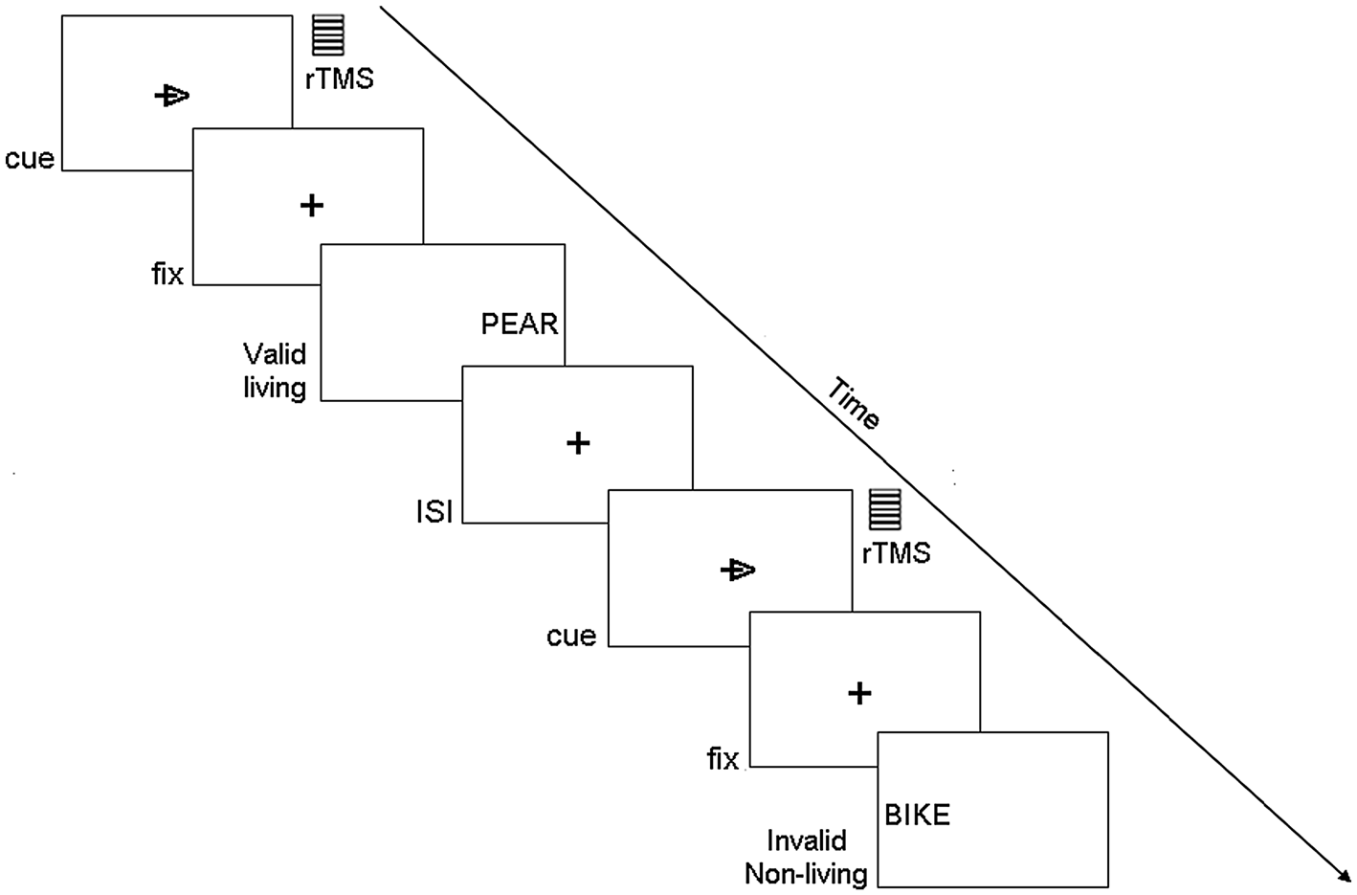
Example of the display sequence in the combined attention-memory task.

However, visual attentional and semantic memory demands often occur simultaneously in everyday life. Although there is a large body of research showing the role of IPS and AG in visual attentional and semantic memory, respectively, no study, to our knowledge, has examined how the brain (and the parietal cortex, in particular) combines these two relevant cognitive demands. It remains largely unknown whether IPS and AG contribute differently to a combined attention-memory task. With this point of view, here, by employing alphanumerical contents, we used a single task (see Fig. 1) implying the information processing of both attention and memory to investigate which part of the parietal cortex has a dominant role in such a combined task.

## Results

### Main analyses

The analyses tested the behavioural effect produced by magnetic stimulation over different parietal sites (IPS and AG) during the execution of a combined attention-memory task.

**Figure 2:**
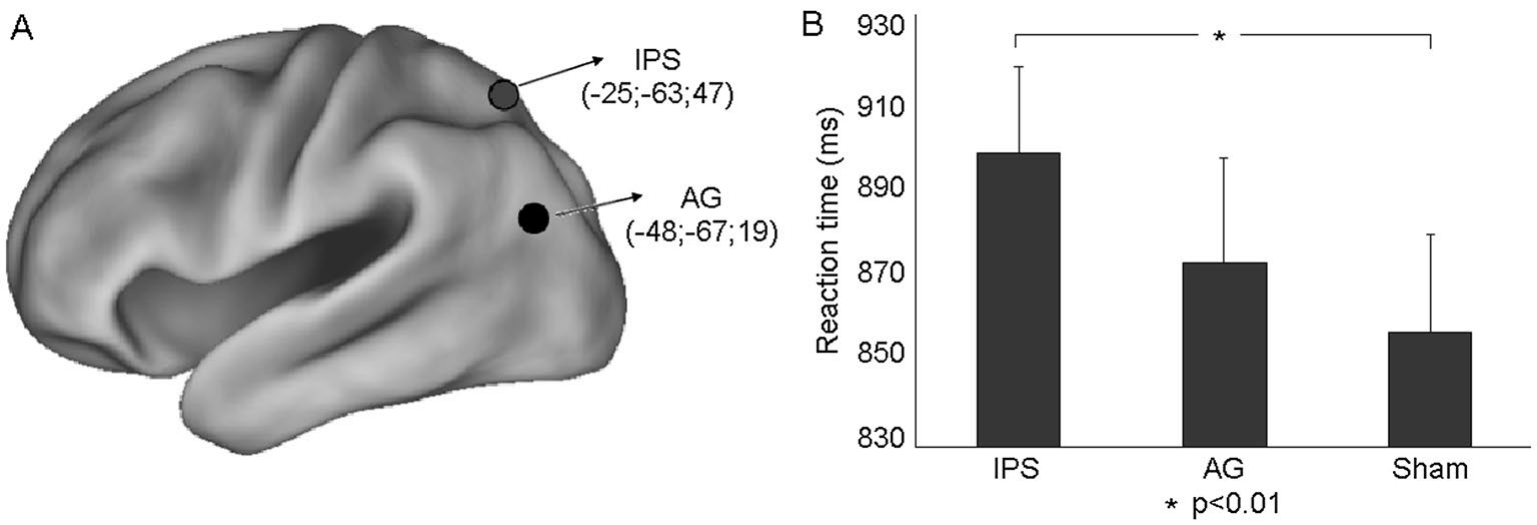
(**A**) Inflated view of left hemisphere atlas brain with regions of DAN (IPS) and DMN (AG) as in meta-analysis by He et al.^14^ and Wirth et al.^10^, respectively. Talairach coordinates (in millimeters) of regions stimulated with rTMS in this experiment are also reported. (**B**) Group means (± standard error, SE) of the RT (ms) for the 3 rTMS Conditions (IPS, AG and Sham). Duncan post hoc tests: one asterisk (p < 0.01).

First, we reported an overall significant main effect of target validity (RTs: valid, 850 ± 21 ms; invalid, 901 ± 23 ms; F(1,17) = 52.6, P = 0.0001) indicating that subjects effectively allocated attention to a specific location of the visual field. More importantly, we also reported a significant main effect of Condition F(2,34) = 4.25, P = 0.023) indicating a more prominent role of IPS. Indeed, relevant Duncan post hoc tests (P < 0.05) showed that the speed of target discrimination during the IPS stimulation (897 ms ± 20) was significantly slowed down as compared to Sham (856 ms ± 22; P = 0.009) but not as compared to AG stimulation (872 ms ± 24; P = 0.09 (Fig. 2B). Moreover, stimulation of AG did not affect behavioural response as compared to Sham (P = 0.27). Of note, it was not observed a statistically significant interaction between condition and target validity (P = 0.737), suggesting that rTMS did not disrupt the observers’ ability to direct spatial attention to the target location.

The ANOVA on accuracy scores did not reveal any significant effect (p = 0.67) indicating a selective interference with the speed of target discrimination.

Furthermore, the analysis to evaluate a possible visual field lateralization showed that the effect of rTMS at different cortical sites was not differential for left (ipsilateral) or right (contralateral) visual field targets (p = 0.147). However, targets presented in the right visual field were identified overall more rapidly than targets presented in the left visual field (left visual field: 888 ± 9 ms; right visual filed: 865 ± 22 ms; F(1,17) = 7.1, P = 0.016), thus reflecting the well-known superiority of the right visual field (left hemisphere) for alphabetical material^15^.

Overall, these results suggest that only IPS stimulation interferes with the performance of the present combined attention-memory task.

### Control analyses

To control the importance of the present sequential evolution of the cognitive demands in the present combined task, and to distinguish between the effects of TMS on isolated tasks versus their combination in direct comparisons, we compared the current results with that of our previous study^12^ in which semantic memory and visuospatial attention tasks were tested in isolation. Specifically, we carried out two separate mixed ANOVAs on RTs of correct responses, with Group (Combined, Attention or Memory) as between-subjects variable, and TMS Condition (AG, IPS, Sham) as within-subject variable. For both analyses we reported the main effect of Group (F(1,34) > 89.41, P < 0.001) since in the combined task subjects took longer to respond compared to both attention (p = 0.001) and memory (p = 0.001) isolated tasks. Moreover, when we compared the current combined task with the earlier semantic memory task we observed an interaction between Group (Combined, Memory) and TMS Condition (AG, IPS, Sham) F(2,68) = 5.18, P = 0.008), showing that the behavioural performance was significantly impaired following stimulation of IPS as compared to AG (P = 0.049) or Sham (P = 0.002), while the opposite pattern, i.e., higher RTs after stimulation of AG as compared to both IPS (P = 0.014) and Sham (P = 0.003), was observed during the memory task. Notably, this pattern of results is in line with what we found in the previous study comparing attention and memory tasks. On the contrary, when we compared the current combined task with the previous attention task we did not report a statistically significant interaction between Group (Combined, Attention) and TMS Condition (AG, IPS, Sham) F(2,68) = 0.86, P =0.43).

## Discussion

The present study examined the causal role of two parietal regions (i.e., IPS and AG) belonging to two distinct brain networks (i.e., DAN and DMN) during the execution of a task combining attentional and semantic memory demands. The results indicate that only the inhibition of IPS affects the behavioural response, thus suggesting its dominant role in such a combined task.

The link between IPS, as part of the DAN, and visuo-spatial attention tasks, as well as the link between AG, as part of the DMN, and semantic memory demand, have been widely demonstrated using a correlative approach (i.e., fMRI)^3,10^. Moreover, previous studies of our group causally confirmed such associations during isolated tasks^12,13,16^). Specifically, only the magnetic stimulation of IPS affects behavioral responses during the execution of a visuo-spatial attention task, whereas only the stimulation of AG interferes during a semantic decision task^12,13^.

Environmental and self-referential processes, such as those induced by visual attentional and semantic memory demands, respectively, are rarely needed in isolation in everyday life. How are these two kinds of cognitive demands flexibly integrated and combined? May the DAN and the DMN directly interact with each other to contribute for example to a combined attention-memory task? The present finding suggests that, compared to a non-active stimulation (i.e., Sham), the behavioral response to a combined attention-memory task is affected only when stimulating the IPS. On the contrary, compared to the Sham condition, the inhibition of AG does not produce interference, despite its well-known role in both semantic memory^9^ and language^7^ processes, which are accounted in the present task. This pattern of results might be explained by the temporal dynamics of the cognitive operations which are engaged to carry out the current task. As matter of fact, to accomplish such a combined task, subjects have to first allocate their visuo-spatial attention and then to process the semantic content of the target. Therefore, it likely that the magnetic stimulation firstly affects the attentional process, hence, it is not surprising to observe a detrimental effect after inhibition of IPS reflecting the sequential evolution of the cognitive demands. Such a conclusion is also confirmed by direct comparison between the present results and those of our previous study in which subjects were asked to perform the two tasks (i.e. attention and memory) separately^12^. In particular when we compared the current combined task with the previous memory task, we reported an interaction similar to what observed in our referenced study between memory and attention tasks. On the contrary, this interaction was not observed when the current combined task was compared to the previous attention task, thus confirming that the present sequential evolution of the cognitive demands induces stronger impairment when magnetic stimulation is delivered over nodes belonging to the attentional network. With this point of view, we suggest that future TMS studies will deeply corroborate such conclusion using experimental paradigm using different timing of stimulation (i.e. before the cue onset and in the period between the cue and the target).

Nevertheless, here we do not report clear statistical differences between IPS and AG, thus suggesting that somehow these regions may have a sort of coactivation in the present combined task. Such coactivation might be characterized by sequential engagement of these parietal regions in different time points along the task execution. On the other hand, based on the lack of difference between the inhibition of the two cortical areas, it can be hypothesized the recruitment of a third-party region to indirectly mediate the dynamic interplay between DAN and DMN as function of task demands. This view comes from a previous EEG-TMS study in which we investigated the changes in the metrics of the resting EEG microstates, which globally represent transient brain activity, after TMS on the IPS and the AG^16^. In that study, we reported that the focal inhibition of both regions resulted in a modification of topography patterns of an EEG microstate previously associated with the cingulo-opercular network (CON), a separate brain system which is thought to be involved in flexible cognitive control^17^, thus supporting the hypothesis that the DAN-DMN interaction is indirectly mediated by a higher-order prefrontal network (CON) involved in the maintenance of the task set.

Nonetheless, the involvement of a third-party network in integrating between environmental and self-referential processes is merely an intriguing hypothesis at the present stage, due to the lack of conclusive experiments in the literature. Hence, we suggest that future neuroimaging studies will directly demonstrate the involvement of specific networks in the combination of the two cognitive processes.

The present findings may form the basis of successful applications, especially in the field of (neuro)psychological (re)habilitation programs. Patients with focal or diffuse brain damage, as well as patients with developmental disorders, often show behavioural impairments in multiple cognitive domains, as well as difficulties in flexibly adapting their cognitive functioning to diverse life situations. This limits the identification of optimal targets for specific cognitive-behavioural treatments or eventually for combined neurostimulation-cognitive approaches. An approach based on dynamic network interactions rather than the role of focal brain regions may help developing new treatment strategy. To conclude, future studies implying also neurophysiological recording will fully shed light on this topic.

## Materials and methods

### Subjects and Stimuli

18 right-handed^18^ volunteers (mean age ± SE = 29.8 ± 5.2 years old, 10 females), with no previous psychiatric or neurological history, participated in the experiment. Informed consent was obtained from all participants according to the Code of Ethics of the World Medical Association, and the Institutional Review Board and Ethics Committee of the University of Chieti. The method of the present study was carried out in accordance with published safety guidelines (see methods section), and the experimental protocol was approved by the Institutional Review Board and Ethics Committee of the University of Chieti. A sensitivity power analysis (GPower software v. 3.1^19^) revealed that our sample size was large enough to detect main effects and interactions of interest with a “small” effect size of 0.18 at an alpha level of p < 0.05 with 0.80 power. Of note, this is consistent with commonly used interpretation referring to effect sizes as “small” (d = 0.2), “medium” (d = 0.5), and “large” (d = 0.8) based on benchmarks suggested by Cohen^20^. The participants were seated on a comfortable reclining armchair and kept their hands on the keyboard. Stimuli were presented on an LCD screen placed at about 80 cm and were generated using E-Prime software v2.0 (Psychological Software Tools, Pittsburgh, PA), and included 200 four-letters Italian nouns, matched for frequency (mean frequency: 13.4).

Subjects were instructed to maintain fixation on a central black cross (subtending 0.2° of visual angle), displayed on a white background at the center of the screen (Fig. 1). During the experimental task, every 4 ± 0.5 s a cue stimulus (a black arrow subtending about 0.2° visual angle and overlapping with the horizontal segment of the fixation cross) was presented for 200 ms duration, randomly cueing either a left (50%) or a right (50%) visual field location. After 2 s from cue onset, the target stimulus (word) was presented for 500 ms at either the cued (valid) or the uncued (invalid) location along the horizontal meridian at 0.7° degrees of visual angle from fixation. The ratio of valid/invalid target was 80/20^21^. The subject's task was to maintain central fixation throughout the trial, covertly pay attention to the location indicated by the cue and make a living/non-living judgment by pressing a corresponding button of the keyboard with their left/right index finger. In this way, subjects performed a semantic judgment during a visuo-spatial task.

Before the experimental sessions, subjects had a long training session (50 trials) to be confident with the task. Then, in each TMS condition we presented 50 trials (40 valid and 10 invalid), so that a single target word was presented only once during the training session (50 trials) and the three experimental conditions (3 conditions × 50 trials = 150 trials).

Subjects were instructed to respond as quick and as accurate as possible. Reaction times and response accuracy were recorded for behavioral analysis.

### TMS procedures and identification of target scalp regions

TMS stimulation was delivered through a focal, figure eight coil, connected with a standard Mag-Stim Rapid 2 stimulator (maximum output 2.2 Tesla). Individual resting excitability threshold for right motor cortex stimulation was preliminarily determined following standardized procedure^22^. The inhibitory rTMS train (i.e., 3 pulses) was delivered simultaneously to the cue onset with the following parameters: 150 ms duration, 20-Hz frequency, and intensity set at 100% of the individual motor threshold. The parameters are consistent with published safety guidelines for TMS stimulation^23^. Of note, previous works from our lab have demonstrated the inhibitory nature of the present stimulation protocol^12,13,16,24,25^.

Each participant performed three conditions, one for each stimulation site, in different blocks, whose order was counterbalanced across subjects. In the two experimental conditions, we stimulated over the left AG and IPS, respectively. In the “Sham” condition, a pseudo rTMS was delivered at scalp vertex; stimulation was ineffective due to the reversed position of the coil with respect to the scalp surface (i.e., the magnetic flux was dispersed to air). Notably, this Sham stimulation produces a similar tactile sensation and alerting (sound, somesthesic stimulation, etc.) to the active rTMS. The location of left AG and IPS was automatically identified on the subject’s scalp using the SofTaxic navigator system (E.M.S. Italy, www.emsmedical.net), which uses a set of digitized skull landmarks (nasion, inion, and two pre-auricular points), and about 40 scalp points entered with a Fastrak Polhemus digitizer system (Polhemus), and an averaged stereotaxic MRI atlas brain in Talairach space^26^. The average Talairach coordinates in the SofTaxic navigator system were transformed through a linear transformation to each individual subject’s scalp. Such method has an error of about 5 mm over a method in which each subject’s own MRI is used for localization^27^. This strategy has been successful in previous rTMS studies^12,24,25,28,29^. A mechanical arm maintained the handle of the coil angled at about 45° away from the midline and the centre of the coil wings was positioned on the scalp, to deliver the maximum rTMS intensity over each site (individual peak of activation). The coordinates of the two cortical areas were based on the previous fMRI study assessing task-evoked activity during spatial attention and semantic memory and were as follows: (i) a region of the DAN: left IPS^14^; (ii) a region of the DMN: left AG^10^ (Fig. 2A).

### Statistical analyses

Statistical analyses were conducted using within-subject ANOVAs for repeated measures. Mauchley’s test was used to evaluate sphericity assumption, Greenhouse–Geisser procedure for correcting the degrees of freedom, and Duncan tests for post hoc comparisons (p < 0.05).

We used RTs of correct responses or percentage of correct responses (Hits) as dependent variables, and TMS Condition (AG, IPS, Sham) and Validity (Valid, Invalid) as the within subject factors. Moreover, to test for possible effect or interaction between visual field and brain area stimulated, we used RTs of correct responses as dependent variables and TMS Condition (AG, IPS, Sham) and Visual field (target stimulus on the right or left side of the screen) as the within-subject factors.

## Data Availability

The datasets used during the current study is available from the corresponding author on reasonable request.
